# Heat stress and heat strain among outdoor workers in El Salvador and Nicaragua

**DOI:** 10.1038/s41370-023-00537-x

**Published:** 2023-04-12

**Authors:** Zoe E. Petropoulos, Sinead A. Keogh, Emmanuel Jarquín, Damaris López-Pilarte, Juan José Amador Velázquez, Ramón García-Trabanino, Magaly Rosario Amador Sánchez, Raúl Guevara, Alexa Gruener, Dustin R. Allen, Jessica H. Leibler, Iris S. Delgado, Michael D. McClean, David J. Friedman, Daniel R. Brooks, Madeleine K. Scammell

**Affiliations:** 1 Department of Environmental Health, Boston University School of Public Health, Boston, MA USA; 2 Agencia para el Desarrollo y la Salud Agropecuaria (AGDYSA), San Salvador, El Salvador; 3 Centro de Hemodiálisis, San Salvador, El Salvador; 4 Emergency Social Fund for Health, Tierra Blanca, El Salvador; 5 Department of Health Sciences, Boston University College of Health and Rehabilitation Sciences, Boston, MA USA; 6 Department of Epidemiology, Boston University School of Public Health, Boston, MA USA; 7 Division of Nephrology, Harvard Medical School and Beth Israel Deaconess Medical Center, Boston, MA USA

**Keywords:** exposure assessment, occupational health, heat strain, heat stress, kidney disease

## Abstract

**Background:**

There is growing attention on occupational heat stress in Central America, as workers in this region are affected by a unique form of chronic kidney disease. Previous studies have examined wet bulb globe temperatures and estimated metabolic rates to assess heat stress, but there are limited data characterizing heat strain among these workers.

**Objective:**

The aims were to characterize heat stress and heat strain and examine whether job task, break duration, hydration practices, and kidney function were associated with heat strain.

**Methods:**

We used data from the MesoAmerican Nephropathy Occupational Study, a cohort of 569 outdoor workers in El Salvador and Nicaragua who underwent workplace exposure monitoring, including continuous measurement of core body temperature (T_c_), heart rate (HR), physical activity, and wet bulb globe temperature (WBGT), over the course of three days in January 2018 - May 2018. Participants represented five industries: sugarcane, corn, plantain, brickmaking, and construction.

**Results:**

Median WBGTs were relatively high (>27 °C) at most sites, particularly when work shifts spanned the afternoon hours (e.g., 29.2 °C among plantain workers). Sugarcane workers, especially cane cutters in both countries and Nicaraguan agrichemical applicators, had the highest estimated metabolic rates (medians: 299–318 kcal/hr). Most workers spent little time on break (<10% of the shift), as determined by physical activity data. Overall, sugarcane workers—particularly those in Nicaragua—experienced the highest T_c_ and HR values. However, a few workers in other industries reached high T_c_ (>39 °C) as well. Impaired kidney function (estimated glomerular filtration rate <90 mL/min/1.73 m^2^) was associated with higher T_c_ and HR values, even after adjustment.

**Significance:**

This is the largest study to-date examining heat stress and strain among outdoor workers in Central America. Workers at sugar companies regularly experienced T_c_ > 38°C (76.9% of monitored person-days at Nicaraguan companies; 46.5% at Salvadoran companies). Workers with impaired kidney function had higher measures of T_c_ and HR.

**Impact statement:**

This study examined levels of occupational heat stress and heat strain experienced among outdoor workers in five industries in El Salvador and Nicaragua. We characterized heat stress using wet bulb globe temperatures and estimated metabolic rate and heat strain using core body temperature and heart rate. Sugarcane workers, particularly cane cutters and Nicaraguan agrichemical applicators, performed more strenuous work and experienced greater levels of heat strain. Impaired kidney function was associated with higher heart rates and core body temperatures.

## Introduction

Occupational heat stress is a significant concern for outdoor workers in physically demanding jobs, particularly under climate change projections that predict increasingly hot ambient conditions [1, 2]. Heavy physical exertion in high heat increases the risk of heat-related illnesses (e.g., heat stroke and heat exhaustion), decline in cognitive function, injury, and death [3–7]. Yet there has been relatively little research on characterizing occupational heat stress and the health effects of chronic exposure [8–10].

In Central America, where outdoor workers and sugarcane workers in particular experience alarming rates of a unique form of chronic kidney disease, interest in occupational heat stress has grown considerably [11–17]. Workers in other agricultural industries and in non-agricultural industries, such as construction and brickmaking, also experience this disease [14, 18, 19] and growing evidence supports the hypothesis that chronic exposure to occupational heat stress plays a role in the disease’s etiology [20, 21].

Occupational heat stress is often characterized using wet bulb globe temperature (WBGT)—an index value combining ambient dry temperature, humidity, wind speed, and solar radiation—compared against established thresholds meant to protect workers from exceeding dangerous core body temperatures (T_c_) [9]. Measuring heat strain—the physiological response resulting from exposure to this heat load—can be more expensive, invasive, or labor-intensive, as it requires use of monitoring devices to measure T_c_, heart rate (HR), and/or change in hydration status/body weight [9].

To date, studies of workers in Central America have relied primarily on a combination of WBGT and work productivity or estimates of metabolic rate, which consistently demonstrate exceedances of health-protective heat stress thresholds [22, 23]. For example, a study among sugarcane workers in El Salvador at different locations and time periods during the 2015 harvest consistently recorded maximum WBGTs above 28 °C [24]. A study among sugarcane workers in Guatemala during the 2017 harvest recorded maximum WBGTs above 35 °C and mean WBGTs above 30 °C [25]. Historical data from Nicaragua demonstrates that heat index values at one sugarcane company met the U.S. Occupational Safety and Health Administration’s high or very high risk criteria on at least 19.6% of harvest days between 2000-2014 [26]. A study of 45 Salvadoran sugarcane cutters found that workers spent the majority of a typical workday above 50% of their estimated maximum HR while being exposed to WBGTs above 26 °C for 79% of the day (maximum WBGT: 32.1 °C) [27].

A handful of studies have monitored T_c_ among agricultural workers in the United States [10] and Mexico using ingestible core temperature sensors, finding that T_c_ occasionally or regularly exceeds 38 °C during the shift (depending on the population) and regularly increases ≥ 1 °C over the course of a work-shift [28–31]. However, differences in job activities, workplace protections [32], hydration practices, co-morbidities, medication use, age, body size, and climate make these findings difficult to extrapolate to working populations in Central America.

This study attempts to address some of these gaps by presenting an analysis of heat stress and heat strain among workers in a variety of industries participating in the MesoAmerican Nephropathy Occupational Study (MANOS). MANOS is a longitudinal occupational cohort study in El Salvador and Nicaragua designed to assess occupational risk factors for kidney injury and kidney disease among outdoor workers [33]. Our primary goal was to characterize heat stress and heat strain among these workers, assessing differences by country, industry, company, and job task. We also sought to understand the potential protective effects of increased hydration and longer breaks. Finally, we wanted to explore whether baseline kidney function was an important factor affecting heat strain.

## Methods

### Study population

The study design and MANOS cohort have been previously described [33]. In brief, MANOS participants (*n* = 569 males) underwent extensive workplace exposure monitoring, including continuous T_c_, HR, physical activity, and WBGT, over three (usually consecutive) days in January 2018–May 2018. Participants represented five industries: corn, plantain, brickmaking, construction, and sugar. We recruited workers from two sugar companies in El Salvador and three in Nicaragua, so the following codes are used for each: SUGAR-E1, SUGAR-E2, SUGAR-N1, SUGAR-N2, and SUGAR-N3.

### Environmental monitoring

WBGT was measured every minute during work shifts using TSI (formerly 3 M) QUESTemp 46 Waterless Wet Bulb Globe Thermometers (TSI Incorporated, Shoreview, MN). Wind speed was also measured, using a TSI air velocity sensor attachment (TSI, Shoreview, MN). Thermometers were mounted on tripods one meter above the ground as close as possible to the participants. If participants moved locations during the work shift, the thermometers were moved to maintain proximity.

### Personal monitoring

T_c_ during the work shift was assessed using wireless ingestible CorTemp® Disposable Temperature Sensors (HQ Inc., Palmetto, FL). Participants were randomly assigned to be monitored during work shifts on Days 1 and 3 or only on Day 2. The CorTemp Data Recorder was worn in a pouch strapped to the small of participants’ backs and recorded T_c_ readings every 10 s.

Physical activity was characterized using an ActiGraph wGT3X BT (ActiGraph, LLC, Pensacola, FL) accelerometer, which captures measured movement at 30 Hz or higher, worn on a belt around the participants’ hips during the work shift on all three days. Polar H7 heart rate monitors (Polar Electro Oy, Kempele, Finland), attached to a strap around the chest below the pectoral muscle, were worn during the work shift on all three days at baseline. Data were collected at a beat-to-beat resolution and transmitted via Bluetooth to the ActiGraph wGT3X BT devices.

Height and weight were measured with a Seca 769 column scale (Seca GmbH, Hamburg, Germany)—before and after each shift for weight, while only once for height. Weight was averaged across all six measurements to determine the participant’s average weight at baseline for Recommended Exposure Limit (REL) calculations and estimated energy expenditure calculations. Differences between pre- and post-shift weight measurements were not used to assess water loss via sweating as the protocol used at each measurement (e.g., clothing and equipment worn) varied considerably.

### Biological samples

Blood samples were collected before and after the shift on the third day only, except for several brick workers (*n* = 29) for whom blood was collected on the first or second day due to unpredictable work schedules. Serum samples from Nicaragua were analyzed at the Ministry of Health’s National Laboratory in Nicaragua and samples from El Salvador were analyzed at Quest Diagnostics in Massachusetts, USA. All samples were analyzed for serum creatinine (IDMS-traceable) to estimate glomerular filtration rate (eGFR) using the CKD-EPI equation [34]. Subsequent serum testing of a random subset of baseline samples (*n* = 50 for each country), conducted at Quest Diagnostics in 2021, confirmed the minimal-to-no difference between laboratories.

### Questionnaires

Questionnaires were administered to participants by trained field team members upon enrollment and at the end of the work shift on each day to capture characteristics of the workday (start and stop time, breaks, hydration practices, medications taken, personal protective equipment worn, and symptoms experienced). Workers reported the job tasks they performed each day, and the study team summarized these tasks into categories (e.g., sugarcane cutting was summarized under “harvesting”). It should be noted that some workers were assigned various tasks throughout the work shift and were summarized into more general job categories.

### Statistical analyses

WBGT, T_c_, HR, and physical activity data for each participant were cropped based on the start and stop times of their work shift. Implausible values for each device (e.g., <30 for HR, < 32 °C for T_c_) were marked as missing and were not included in calculations. WBGT, T_c_, HR, or physical activity data with more than 50% of any given work shift missing were excluded from relevant analyses (*n* = 123 person-days for HR; 40 for physical activity; 141 for WBGT; 60 for T_c_).

All MANOS participants were outdoor workers, so the WBGT formula for outdoor settings was used. The first 10 min of each WBGT dataset was removed, prior to cropping at the work shift, to account for the stabilization period defined by the manufacturer [35]. When available, two thermometers were used simultaneously and the values from each device were averaged at each time point. Effective WBGT (WBGT_eff_) was calculated by adding a clothing adjustment factor of 0.5 °C for agrichemical applicators in Nicaragua, who wore polypropylene and plastic coveralls [9, 36]. Data were smoothed using a 20-minute rolling average. The REL—the recommended heat stress exposure threshold defined by the National Institute of Occupational Safety and Health (NIOSH)—was calculated for each participant using their average body weight and estimated average metabolic rate (kcalories/hour) derived from physical activity data (described below). Minutes above REL and percent of the work shift above REL were calculated for each participant on each shift. The heat index was derived from dry temperature and humidity using the National Weather Service formula through the *weathermetrics* package in R [37, 38].

For accurate T_c_ readings, CorTemp® sensors need to pass through the digestive system to the small intestine, which requires swallowing the sensor several hours before monitoring and ideally eating/drinking something with the sensor. If the sensor is too high in the digestive tract, the T_c_ data can be influenced by the consumption of liquids and foods resulting in data reflecting a “bouncing ball” effect [39]. Despite study protocol stating that sensors should be swallowed the night before the monitoring workday, it was often difficult to put this into practice. Workers and investigators had concerns about bowel movements prior to the work shift and ingestion protocol compliance, therefore it was not uncommon for the sensors to be swallowed the morning of monitoring. The number of hours before the work shift that the sensor was swallowed varied widely, with distinct patterns by country, industry, and work site due to logistics. For this reason, the number of hours before the shift the sensor was swallowed was estimated to examine the effect of this variation. In addition to the sensor being higher in the digestive tract than desired, other issues can cause nonsensical T_c_ data. For instance, workers standing close to one another may cause interference in the transmission of T_c_ data to the correct data recorder and the presence of two sensors in the body (e.g., worker thinking they had excreted the first sensor) may produce unusable data. T_c_ data for each participant were carefully examined to identify such files. A script in R flagged T_c_ data afflicted by the “bouncing ball” effect—but otherwise deemed usable—and removed portions of the T_c_ data that were unrealistic based on the magnitude of the slope between neighboring points 1, 2, and 3 points away. Criteria for removal were as follows:

1. The average slope between a given point and its neighboring points on either side was > 2*SD away from the mean of all slopes of that window size (i.e., 1 point away, 2 points away, etc.) and the value of the temperature at that point was > 2*SD away from the mean of all temperature values for that individual, or
2. The absolute slope between a given point and its neighboring points was greater than the equivalent of a 2 °C change over 15 min.

All T_c_ data were then smoothed using local regression (LOESS) using a 25% smoothing span.

Vector magnitude (VM)—defined as the square root of the sum of the squares of the counts for each of the three axes measured by the accelerometers—was used to estimate energy expenditure in kilocalories at each minute interval using the 2011 Freedson VM3 equation [40] combined with the 1998 Williams Work-Energy Equation [41]:

$$	{{{{{{{\mathbf{if}}}}}}}}\;{{{{{{{\mathbf{CPM}}}}}}}}\, > \,{{{{{{{\mathbf{1951}}}}}}}}\;{{{{{{{\mathbf{then}}}}}}}}\\ 	{{{{{{{\mathbf{kcals}}}}}}}}/{{{{{{{\mathbf{min}}}}}}}} = {{{{{{{\mathbf{0}}}}}}}}.{{{{{{{\mathbf{00094}}}}}}}}\, \times {{{{{{{\mathbf{CPM}}}}}}}} + {({{{{{{{\mathbf{0}}}}}}}}.{{{{{{{\mathbf{1346}}}}}}}}\, \times {{{{{{{\mathbf{BM}}}}}}}} - {{{{{{{\mathbf{7}}}}}}}}.{{{{{{{\mathbf{37418}}}}}}}}} )\\ 	{{{{{{{\mathbf{else}}}}}}}}\;{{{{{{{\mathbf{kcals}}}}}}}}/{{{{{{{\mathbf{min}}}}}}}} = {{{{{{{\mathbf{CPM}}}}}}}}\, \times {{{{{{{\mathbf{0}}}}}}}}.{{{{{{{\mathbf{0000191}}}}}}}} \times {{{{{{{\mathbf{BM}}}}}}}}$$

where BM is body mass in kilograms and CPM is the counts per minute (i.e., vector magnitude at a minute interval). Vector magnitude was also used to determine when participants were on break or otherwise performing limited physical activity, using the threshold of VM < 150 CPM [42].

LOESS regression with a 10% smoothing span was used to smooth HR data. Maximum HR (HR_max_) was calculated using the formula 220-age and percent of HR_max_ at each minute interval was calculated using the smoothed HR at that interval.

Multivariable linear regression models were used to examine the associations between job task, hydration practices, break duration, and baseline kidney function and maximum T_c_ experienced during the work shift, controlling for confounders which were selected using a literature review of relevant research and a directed acyclic graph. Mixed effects models with a random intercept and random slope for day were used for modeling the median percent of HR_max_ experienced during each shift. Pre-shift eGFR was used to assess kidney function at baseline using the following categories: < 60, 60–90, and >90 mL/min/1.73 m^2^. Participants with pre-shift eGFR <60 mL/min/1.73 m^2^ (*n* = 53) were considered to have impaired kidney function and were removed from all models, except for those examining the effects of kidney function on measures of heat strain. Data for overnight shifts (*n* = 48 person-days; 2.8% of all person-days) were removed from all models, as were any person-days for which the monitoring data captured < 50% of shift. Models examining electrolyte solution were restricted to Nicaraguan sugar workers, as they were the only workers reporting consumption of this beverage.

Analyses were performed using SAS Version 9.4 and R Version 3.6.1 (The R Foundation for Statistical Computing, www.r-project.org) [43].

## Results

The study team was able to capture HR and accelerometer data for the entirety of most participants’ work shifts (Table S1). This was also true for WBGT monitoring in El Salvador, but less so in Nicaragua (median percent of shift captured: 61-84%). WBGT monitoring was absent during many brick workers’ shifts, due to only having two thermometers and multiple, concurrent work sites. T_c_ monitoring covered at least 80% of the work shift for most participants on their respective T_c_ monitoring day(s), even after data cleaning. Other variables (e.g., self-reported hydration) were captured for most every work shift for every participant.

The mean age for participants in most industries was 28-31 years (Table 1). Participants across industries were of similar height. Average weights varied from the lowest mean weight among SUGAR-E2 (64.7 kg ± 9.6) to the highest among construction (73.5 kg ± 11.8).

**Table 1:** Summary statistics for the participant, work monitoring, and biomarker data, by industry/sugar company.

	El Salvador				Nicaragua				
	Sugar-E1	Sugar-E2	Corn	Cons	Sugar-N1	Sugar-N2	Sugar-N3	Brick	Plan
Total number of participants	55	56	110	58	22	52	50	107	59
**Work shift characteristics**									
Mean Work Shift Duration (SD) (hours)	4.6 (3.3)	5.0 (2.2)	3.2 (1.5)	9.3 (1.1)	3.7 (0.3)	4.2 (1.4)	5.1 (1.9)	6.6 (2.9)	7.2 (3.2)
Typical Shift Start Time (1^st^ quartile-3^rd^ quartile)	6:15-7:15	6:50-7:30	6:00-6:45	7:00-7:20	6:30-7:00	6:30-7:00	7:10-7:55	2:17-6:49	6:20-6:40
Typical Shift Stop Time (1^st^ quartile-3^rd^ quartile)	8:00-16:00	10:00-14:20	8:40-10:00	15:38-17:00	10:15-10:35	9:40-12:00	10:57-14:15	9:08-12:00	11:30-17:00
**Environmental heat**									
Median WBGT (MAD) (°C)	26.0 (2.1)	28.9 (1.1)	27.1 (1.5)	28.9 (0.52)	28.3 (0.23)	26.4 (0.98)	27.1 (0.55)	27.0 (1.7)	29.2 (0.83)
Maximum WBGT (MAD of maximums) (°C)	30.5 (3.1)	32.4 (1.6)	34.6 (3.0)	32.8 (0.56)	29.3 (0.21)	30.3 (1.9)	30.8 (0.7)	32.7 (2.4)	33.5 (1.6)
Median Heat Index (MAD) (°C)	28.5 (3.5)	32.5 (1.3)	29.8 (2.7)	33.0 (1.1)	31.0 (0.53)	30.7 (0.89)	31.7 (0.98)	31.1 (2.7)	36.1 (1.5)
Number of person-days with WBGT_eff_ exceeding REL_accel_ at least 25% of shift (% of person-days)	7 (4.3%)	58 (35%)	20 (6.3%)	11 (6.3%)	52 (79%)	23 (15%)	42 (28%)	0 (0%)	15 (10%)
**Core body temperature**									
Median T_c_ (MAD) (°C)	37.7 (0.31)	37.7 (0.31)	37.4 (0.35)	37.5 (0.26)	37.7 (0.66)	37.9 (0.31)	37.8 (0.37)	37.5 (0.36)	37.6 (0.30)
Maximum T_c_ (MAD of maximums) (°C)	40.1 (0.30)	39.1 (0.23)	38.9 (0.37)	40.2 (0.25)	39.8 (0.53)	39.3 (0.26)	39.3 (0.27)	39.1 (0.22)	39.2 (0.40)
*(Sensor swallowed* > *3* *hours before shift only)* Maximum T_c_ (MAD) (°C)	40.1 (0.24)	38.8 (0.21)	38.9 (0.36)	38.6 (0.20)	39.5 (0.15)	39.3 (0.27)	38.8 (0.21)	39.1 (0.25)	39.2 (0.42)
*(Sensor swallowed* > *3* *hours before shift only)* Median T_c_ (MAD) (°C)	37.7 (0.32)	37.7 (0.30)	37.8 (0.52)	37.6 (0.17)	37.8 (0.75)	37.9 (0.31)	37.8 (0.35)	37.5 (0.34)	37.5 (0.32)
Number of person-days with T_c_ > 38 °C at least 5 minutes (% of person-days with T_c_)	29 (41%)	37 (52%)	37 (22%)	23 (28%)	16 (73%)	54 (81%)	30 (73%)	25 (21%)	12 (35%)
Number of person-days with T_c_ > 38.5 °C at least 5 minutes (% of person-days with T_c_)	4 (6%)	3 (4%)	9 (5%)	4 (5%)	7 (32%)	7 (10%)	6 (15%)	4 (3%)	2 (6%)
Median percent of shift T_c_ > 38 °C	0%	4%	0%	0%	21%	29%	16%	0%	0%
Median cross-shift T_c_ increase (MAD) (°C)^1^	0.56 (0.34)	0.65 (0.45)	0.62 (0.50)	0.62 (0.32)	1.3 (0.48)	0.98 (0.38)	1.1 (0.37)	0.73 (0.52)	0.75 (0.36)
**Metabolic Rate and Time on Break**									
Median Metabolic Rate (MAD) (kcal/hour)	224.5 (207.7)	195.0 (115.4)	101.5 (54.7)	82.5 (52.0)	402.1 (56.8)	291.3 (80.0)	291.6 (74.3)	174.9 (82.8)	102.8 (93.1)
Median percent shift VM < 150 CPM (MAD)	4.8% (7.1%)	12% (7.2%)	4.1% (6.0%)	19% (10%)	5.3% (4.5%)	4.8% (3.7%)	6.9% (3.7%)	6.1% (6.1%)	21% (12%)
**Heart rate**									
Median % HR_max_ (MAD)	56% (12%)	54% (8.5%)	54% (6.4%)	50% (8.1%)	66% (7.2%)	61% (6.6%)	62% (5.6%)	51% (7.3%)	47% (7.7%)
Number of person-days with HR > 75% HR_max_ ≥ 25% shift (% of person-days)	11 (6.8%)	3 (1.9%)	12 (3.7%)	28 (16%)	20 (35%)	8 (6.0%)	23 (16%)	6 (2.0%)	5 (3.5%)
Median cross-shift HR increase (MAD) (bpm)	32 (20)	37 (20)	30 (20)	27 (21)	62 (26)	49 (23)	43 (26)	25 (22)	24 (23)
**Baseline Kidney Function**									
Number of participants with eGFR < 60 (%)	9 (16%)	13 (23%)	12 (11%)	2 (3%)	0 (0%)	1 (2%)	0 (0%)	11 (10%)	5 (9%)
Number of participants with eGFR 60-90 (%)	1 (1.8%)	8 (14%)	8 (7.2%)	3 (5.2%)	9 (41%)	7 (14%)	1 (2%)	13 (12%)	10 (17%)

MAD Median absolute deviation, REL_accel_ Recommended Exposure Limit for wet bulb globe temperatures determined by accelerometer-based estimates of metabolic rate, VM Vector magnitude, CPM Counts per minute, bpm beats per minute, eGFR estimated glomerular filtration rate, CONS construction, PLAN plantain.

^1^ – Cross-shift increases were calculated by subtracting the first non-missing reading from the maximum value recorded.

Construction and plantain workers had, on average, the longest shifts (9.3 h and 7.2 h, respectively); however, some oven burners in brickmaking had shifted as long as 24–30 h and some plantain workers had much shorter shifts (2–4 h) (Table 1). Sugarcane and corn workers had shorter shifts, with mean durations of 3–5 h.

Higher WBGT values were observed at construction, plantain, and two sugar sites (SUGAR-E2 and SUGAR-N1). Construction and plantain workers have more frequent afternoon work hours when the temperatures are higher (Table 1). The highest median T_c_ values were observed at SUGAR-N2. All industries except corn had a least one worker with a Tc reading above 39 °C. After excluding individuals who swallowed the T_c_ sensor <3 h before the shift, SUGAR-E1, SUGAR-N1, and SUGAR-N2 had the most extreme maximum T_c_ readings, with SUGAR-E1 at 40.1 °C being the highest. Nicaraguan sugar companies had the highest rates of workers’ T_c_ exceeding 38 °C and 38.5 °C during the work shift, with the majority of work shifts observed at those three companies and SUGAR-E2 involving T_c_ > 38 °C for ≥ 5 min. Workers at the Nicaraguan sugar companies were also more likely to spend a significant percentage of their work shift with T_c_ > 38 °C--with the medians ranging from 16%-29% of the work shift—and had the highest median cross-shift increases in T_c_, ranging from 0.98 to 1.3 °C.

Nicaraguan sugarcane workers engaged in the most strenuous work (based on median estimated metabolic rates), while workers in corn, construction, and plantain had much lower median metabolic rates (Table 1). Similarly, the sugarcane workers in Nicaragua also experienced higher percentages of their HR_max_ during the work shift and higher cross-shift increases in HR. Over a third of person-days observed at SUGAR-N1 (35%) involved workers spending at least a quarter of the work shift above 75% of their estimated HR_max_. According to the accelerometer data, construction and plantain workers spent the greatest percent of their work shift on break (~20% for each), followed by SUGAR-E2 (12%). The average percent of shift on break at other sugar companies and other industries was 4–7%.

Based on RELs derived from estimated metabolic rates, experiencing WBGT temperatures above respective RELs for at least a quarter of the work shift was most common at SUGAR-N1, SUGAR-E2, and SUGAR-N3 (Table 1). Approximately 4 of every 5 person-days observed at SUGAR-N1 involved effective WBGTs above the REL for at least a quarter of the shift.

Self-reported total water consumption was mostly consistent across industries and companies despite different shift durations, with workers reporting 3-4 L during the work shift, except for SUGAR-E2 (median: 5.5 L) and SUGAR-N3 (median: 8.0 L). Most participants did not report consuming electrolyte solution during the work shift, except for Nicaraguan sugarcane workers, who reported consuming 0.6–2.0 L per shift, on average. The electrolyte solution was provided to these workers by their employer.

Due to pre-harvest serum creatinine screening at the Nicaraguan sugar companies, there were far fewer participants with a baseline eGFR <60 mL/min/1.73 m^2^ (range: 0–2%) compared to other sites (range: 3–23%). However, there were participants at these companies with eGFR between 60 and 90 ml/min/1.73 m^2^, with an especially large percentage at SUGAR-N1 (41%, *n* = 9).

**Fig. 1:**
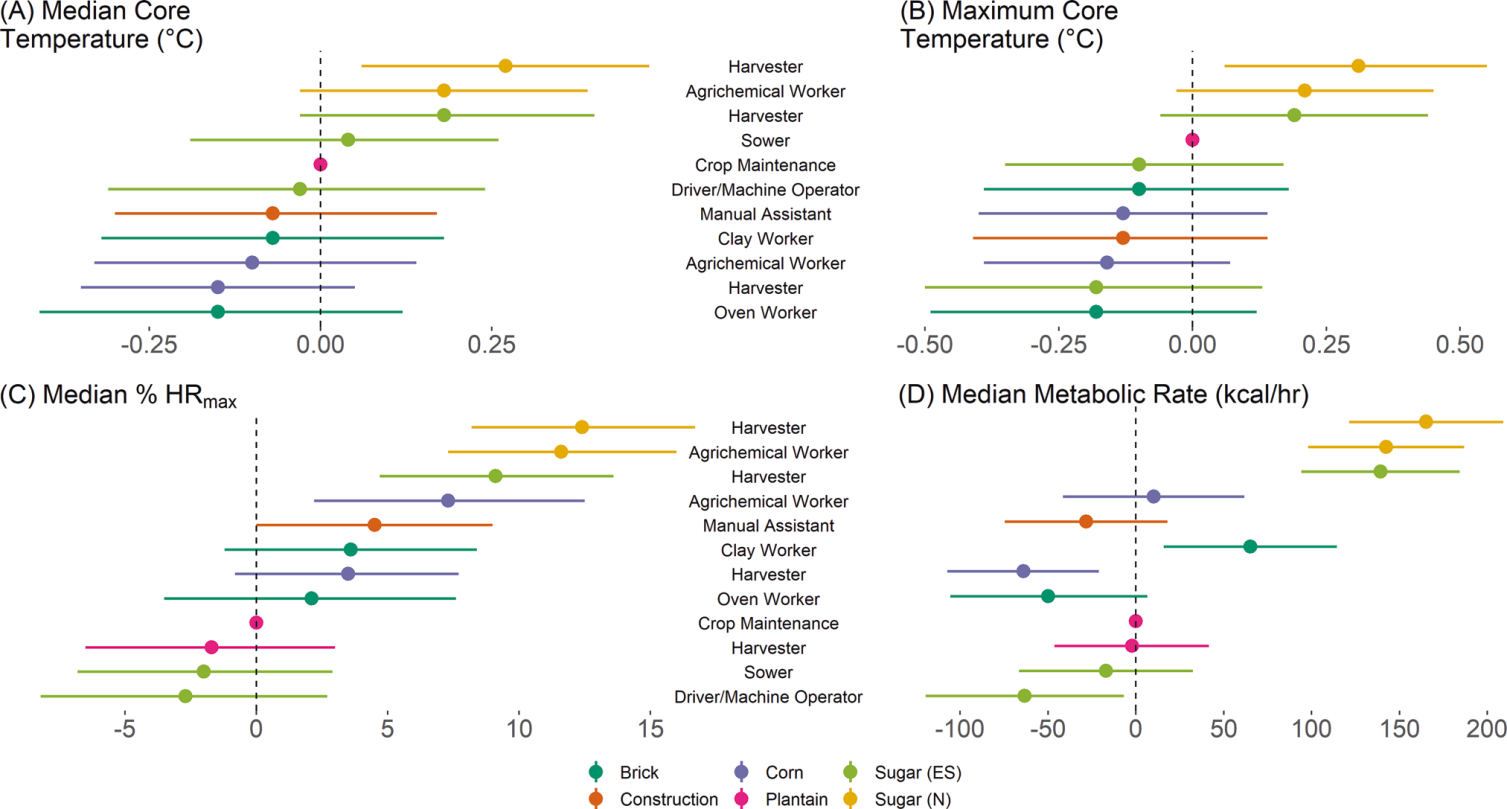
Adjusted core temperature, heart rate, and metabolic rate, by job task and industry, relative to reference group. Parameter estimates and 95% confidence intervals for job task in regression models for **A** median Tc, **B** maximum Tc, **C** median % HRmax, and **D** median metabolic rate, after adjusting for shift duration, median WBGT, and kidney function.

For T_c_ and HR, Nicaraguan sugar agrichemical applicators and sugarcane harvesters in both countries consistently had the highest readings after adjusting for shift duration, median WBGT, and kidney function (Fig. 1). These were the same workers with the highest estimated metabolic rates, after adjusting for shift duration and median WBGT. These findings were all largely seen before adjustment as well (Table S2). The summary statistics for shift characteristics, monitoring data, and self-reported hydration by job task can be found in the supplemental material (Tables S2-S4).

Most sugarcane workers consumed at least 1 L of water per hour, except among the lower-intensity jobs of the driver, machine operator, and supervisor/irrigator, for whom the median was around 0.5 L/h (Table S2). Workers in brick, construction, and plantain reported lower water consumption rates (median rates: 0.3–0.6 L/h) than most sugarcane workers (Tables S3-S4). Workers in corn reported higher, but more variable water consumption (median rates: 0.9–1.9 L/h) (Table S4).

Increased consumption of water during the shift and time spent at a low vector magnitude were not associated with maximum T_c_ during the shift (Table 2). Consumption of electrolyte solution was positively associated with maximum T_c_ (increase of 0.15 °C per L consumed; 95% CI: 0.04, 0.26). Workers with lower eGFRs (<60 and 60–90 mL/min/1.73 m^2^) experienced higher maximum T_c_ when compared to workers with eGFR > 90 (0.20 °C and 0.11 °C higher T_c_, respectively), after controlling for industry, job task, and age.

**Table 2:** Summary of multivariable linear regression models examining risk/protective factors associated with maximum T_c_.

	Crude Models		Adjusted Model 1^a^		Adjusted Model 2^b^	
	B (95% CI)	p-val	B (95% CI)	p-val	B (95% CI)	p-val
**Water Consumption Rate (per 0.6 L/h)**	0.02 (-0.01, 0.05)	0.25	0.00 (-0.03, 0.04)	0.82	-0.02 (-0.06, 0.01)	0.22
a – Adjusted for industry/company, job task, shift duration, and consumption of other beverages b – Additionally adjusted for median WBGT, mean metabolic rate, and percent shift VM < 150 cpm						
**Total Electrolyte Solution Consumption** **(per 1 L)**	0.06 (-0.02, 0.14)	0.14	0.16 (0.06, 0.27)	0.002	0.15 (0.04, 0.26)	0.01
a – Adjusted for industry/company, job task, shift duration, and consumption of other beverages b – Additionally adjusted for median WBGT, mean metabolic rate, and percent shift VM < 150 cpm						
**Percent Shift Vector Magnitude < 150 cpm** **(per 10% increase)**	-0.03 (-0.01, 0.00)	0.07	-0.02 (-0.06, 0.02)	0.24	0.00 (-0.04, 0.04)	0.93
a – Adjusted for industry/company, job task, and shift duration b – Additionally adjusted for total liquid consumption, median WBGT, and mean metabolic rate						
**eGFR < 60 ml/min/1.73 m**^**2**^ **(vs > 90)**	0.23 (0.11, 0.34)	0.0002	0.20 (0.09, 0.31)	0.001		
**eGFR 60–90 ml/min/1.73 m**^**2**^ **(vs > 90)**	0.14 (0.01, 0.27)	0.03	0.11 (-0.01, 0.22)	0.07		
a – Adjusted for industry/company, job task, and age.						

WBGT wet bulb globe temperature, VM vector magnitude, cpm=counts per minute, L/hr liters per hour.

Spending a greater portion of the work shift on break (as determined by physical activity data) was associated with lower heart rates (as % of HR_max_); an increase of 10% of the work shift spent on break was associated with an absolute decrease in median % HR_max_ of 1.5% (95% CI: -2.1%, -0.85%) (Table 3). Consumption of water and electrolyte solution was associated with greater median % HR_max_ (+0.46% and +1.2%, respectively). Low baseline eGFR (< 60 and 60–90) was associated with higher median % HR_max_ (1.7 and 2.9 % points higher, respectively, compared to >90).

**Table 3:** Summary of mixed effects models examining risk/protective factors associated with median % HR_max_.

	Crude Models		Adjusted Model 1^a^		Adjusted Model 2^b^	
	B (95% CI)	p-val	B (95% CI)	p-val	B (95% CI)	p-val
**Water Consumption Rate (per 0.6 L/h)**	1.6 (1.1, 2.0)	<.0001	1.2 (0.65, 1.7)	<.0001	0.46 (-0.04, 0.97)	0.07
a – Adjusted for industry/company, job task, shift duration, and consumption of other beverages b – Additionally adjusted for median WBGT, mean metabolic rate, and percent shift VM < 150 cpm						
**Total Electrolyte Solution Consumption** **(per 1 L)**	1.6 (0.67, 2.6)	0.001	1.4 (0.42, 2.5)	0.007	1.2 (0.34, 2.1)	0.009
a – Adjusted for industry/company, job task, shift duration, and consumption of other beverages b – Additionally adjusted for median WBGT, mean metabolic rate, and percent shift VM < 150 cpm						
**Percent Shift Vector Magnitude < 150 cpm** **(per 10% increase)**	-3.2 (-3.7, -2.7)	<0.0001	-2.8 (-3.4, -2.2)	<0.0001	-1.5 (-2.1, -0.85)	<0.0001
a – Adjusted for industry/company, job task, and shift duration b – Additionally adjusted for total liquid consumption, median WBGT, and mean metabolic rate						
**eGFR < 60 ml/min/1.73 m**^**2**^ **(vs > 90)**	2.2 (-0.42, 4.9)	0.10	1.7 (-0.40, 3.9)	0.11		
**eGFR 60–90 ml/min/1.73 m**^**2**^ **(vs > 90)**	4.2 (1.5, 6.8)	0.002	2.9 (0.87, 5.0)	0.005		
a – Adjusted for industry/company, job task, age, and mean metabolic rate.						

Parameter estimates are reported as absolute increase/decrease in HR_max_ percentage points, not relative change in percentage of HR_max_.

WBGT wet bulb globe temperature, VM vector magnitude, cpm counts per minute, L/h liters per hour.

## Discussion

We found that among MANOS participants, workers in the sugarcane industry, especially in Nicaragua, seem to be performing the most physically intense work, working in ambient conditions above recommended guidelines, and experiencing the greatest levels of heat strain.

The median estimated metabolic rates were much higher among sugarcane workers (range: 195-402 kcal/hour) than the other industries (range: 83-175 kcal/hour), with the highest at SUGAR-N1, which was comprised entirely of agrichemical applicators (whose median metabolic rate was >3x that of Salvadoran agrichemical applicators, likely because of the faster pace these applicators must keep in order to cover the assigned acreage). Despite typically avoiding work during the afternoon hours, high median WBGTs were observed at SUGAR-E2 and SUGAR-N1 (28.9 °C and 28.3 °C, respectively), similar to the median WBGTs observed at construction and plantain (28.9 °C and 29.2 °C), the industries most likely to work in the afternoon. The high WBGT temperatures and metabolic rates observed at SUGAR-E2 and SUGAR-N1 contributed to the high percentages of monitored work shifts during which the WBGT_eff_ exceeded the estimated REL for at least 25% of the shift (35% and 79% of shifts, respectively).

These factors translated into consistent findings for heat strain—with workers at the Nicaraguan sugar companies experiencing the highest measures of T_c_ and % HR_max_. Despite working in relatively cooler WBGTs, workers at SUGAR-N2 had the highest median T_c_ at 37.9 °C. Interestingly, the median T_c_ values observed at sugar companies were consistently higher than those observed in a small pilot study of migrant agricultural workers in northern Mexico in June/August [31], which were more closely aligned with the values we observed for corn and plantain workers. Extreme maximum T_c_ values (>39 °C) were observed at all industries, with the highest in each country being observed at a sugar site. One-third of T_c_-monitored SUGAR-N1 workers exceeded 38.5 °C for at least 5 minutes during the shift, similar to a study of workers in Florida which found that a quarter of crop workers reached the same threshold [30].

The median % HR_max_ experienced during the work shift by Nicaraguan sugarcane workers ranged from 61-66%, with SUGAR-N1 the highest. This was comparable among Salvadoran sugarcane harvesters (median: 60%). These are higher than the median value reported by Lucas et al. 2015 [27] among Salvadoran cane cutters—54%. While that study did employ a different approach for estimating HR_max_, this difference in methodology would only affect individuals > 40 years old, so is an unlikely explanation for the difference between studies. The median HR values observed among Nicaraguan sugarcane workers in MANOS are comparable with those measured by the Adelante Initiative in a similar subset of workers [44].

Sugarcane workers, on average, reported consuming the most water, with most workers consuming more than 1 L/h. Workers in less strenuous jobs at sugar sites reported lower consumption rates. We know that Nicaraguan sugar companies have instituted workplace protections to reduce heat strain, which includes obligatory hydration with water (at least 1 L/hour) and electrolyte solution. These hydration requirements are reflected in the self-reported hydration data from workers. Despite any policies currently in place at the sugar sites, we observed relatively infrequent periods of low movement (VM < 150 cpm), with an average of 5–7% of the shift spent “on break” at the Nicaraguan sugar companies.

Increased time spent on break and consumption of water did not appear to have a protective effect on elevated T_c_ in our models. This may be due to exposure misclassification for both variables and/or incomplete control of confounding. We are not advocating for sugar companies to relax any hydration policies based on this study, especially since intervention studies provide some evidence that increased hydration along with other interventions during the work shift may reduce declines in kidney function [24, 45]. The finding that electrolyte solution consumption was positively associated with T_c_ may be a result of incomplete control for confounding by metabolic rate. The most interesting finding for the T_c_ and % HR_max_ models was the evidence that workers with low eGFR have higher average T_c_ and HR values. These findings suggest that there could be a cycle of impaired kidney function leading to impaired thermoregulation (potentially via impaired water retention), which could increase an individual’s risk of acute kidney injury.

Building a causal model for HR in this study is difficult as HR increases both when an individual is working strenuously (to carry oxygen to muscles) and when an individual is exposed to a high heat load (because sweating and peripheral vasodilation lead to a loss of blood volume/low blood pressure, which results in a higher HR to maintain cardiac output). We found that spending more time on break (as determined by physical activity) was associated with a lower average % HR_max_, which is consistent with both mechanisms. However, increased consumption of water and electrolyte solution were positively associated with %HR_max_. This finding may be a result of incomplete control for confounding by estimated metabolic rate or urine concentrating ability.

There are a few limitations with these data that require consideration. For one, we only conducted three days of monitoring (fewer for T_c_) and ambient conditions likely varied by industry and month of year because of our monitoring schedule (i.e., each industry and sugar company was visited sequentially, not concurrently). Work activities and conditions may not have been representative of typical workdays for a few reasons. Some workers perform varied tasks, which typically change day-to-day. We also cannot rule out the possibility that our study team’s presence affected the shift duration or job tasks performed on the days of monitoring. We do know from prior research and anecdotal knowledge that sugarcane cutters’ shift durations have typically been longer than what we observed for MANOS. However, it is worth noting that we still found high T_c_ among these workers, indicating that typical values for these sugarcane workers could potentially be even higher.

WBGT monitors were not always available during oven worker shifts, and when they were, they were not always able to be placed close to workers because of potential damage to the equipment caused by the heat of the ovens. Therefore, the WBGT data that was collected for these workers may not fully capture their exposure to environmental heat. Additionally, many of the oven workers worked overnight shifts, which may skew their summary statistics given lower temperatures and less physical activity during the overnight hours.

Accelerometer data were used to estimate energy expenditure, which likely introduced some misclassification as efficiency varies between individuals. Specifically for sugarcane cutters, we may have underestimated metabolic rate due to the placement of accelerometers on the hip instead of the arm given that cane cutting is an arm-intensive activity. However, cane cutters still had some of the highest estimated metabolic rates, and these would be considered conservative estimates. T_c_ sensors were not always swallowed early enough for high-quality data and this varied by industry/company. However, precautions were taken to avoid using inaccurate data. We also relied on some self-reported data (e.g., hydration), which means we should expect some misclassification and a potential bias in our model results for these variables.

Future studies should determine whether these measures of heat strain correlate with outcomes of interest, such as acute kidney injury. These data can also be used to determine which WBGT thresholds/screening guidance should be used for preventing adverse health outcomes (e.g., kidney injury) in these workers if exposure-response estimates are established.

We leveraged data from a large cohort study to describe levels of occupational heat stress and heat strain among outdoor workers in Central America—a topic of much interest given climate change and the current epidemic of chronic kidney disease in the region. We found that sugarcane cutters and agrichemical applicators, particularly those in Nicaragua, perform physically intense work in hot conditions and occasionally experience very high T_c_. These workers spend relatively little time on break according to accelerometer data. We found that workers in other industries also occasionally experience high T_c_. Regardless of whether heat stress is causally involved in MeN or related to its progression following preexisting kidney impairment, we identified levels of heat strain that warrant follow-up in order to prevent heat-related illness and injury in this population.

## Supplementary information

Supplemental Material

## Data Availability

The datasets generated and analyzed for the current study are not publicly available because they are being analyzed in relation to outcomes. When these analyses are complete, we intend to make both exposure and outcome data available in a public repository. In the meantime, we will make the exposure data available upon reasonable request made to the last author. Additionally, we hope the supplemental tables will give readers a more complete understanding of the data.
