# Supplementary material for: Heat stress and heat strain among outdoor workers in El Salvador and Nicaragua
Source: J Expo Sci Environ Epidemiol. 2023 Apr 12;33(4):622–30. doi: 10.1038/s41370-023-00537-x (PMC10403352; doi:10.1038/s41370-023-00537-x)
Supplement: Supplementary file 1 — Supplemental Material [file 41370_2023_537_MOESM1_ESM.docx]

**Supplemental Material**

**Table of Contents**

1. Table S1. Summary of collected monitoring and biomarker data, by industry/company
2. Table S2. Summary statistics for shift characteristics, work monitoring, and biomarker data, by job task within sugar industry
3. Table S3. Summary statistics for monitoring and biomarker data, by job task within non-agricultural industries.
4. Table S4. Summary statistics for monitoring and biomarker data, by job task within non-sugar agricultural industries.

Table S1. Summary of collected monitoring data, by industry/company

|  | **El Salvador** | | | | **Nicaragua** | | | | |
| --- | --- | --- | --- | --- | --- | --- | --- | --- | --- |
|  | **SUGAR-E1** | **SUGAR-E2** | **CORN** | **CONS** | **SUGAR-N1** | **SUGAR-N2** | **SUGAR-N3** | **BRICK** | **PLAN** |
| Total number of participants | 55 | 56 | 110 | 58 | 22 | 52 | 50 | 107 | 59 |
| Total person-days | 165 | 168 | 330 | 174 | 66 | 156 | 150 | 321 | 177 |
| Person-days of WBGT monitoring | 164 | 168 | 318 | 174 | 66 | 155 | 149 | 106 | 143 |
| Median % of Work Shift with WBGT data | 100% | 98% | 100% | 93% | 68% | 84% | 70% | 79% | 61% |
| Person-days with coverall use | 0 | 0 | 0 | 0 | 66 | 60 | 59 | 0 | 39 |
| Person-days of T_c_ monitoring | 71 | 71 | 172 | 81 | 22 | 67 | 41 | 119 | 34 |
| Median % of Work Shift with Tc data | 80% | 87% | 85% | 69% | 74% | 89% | 85% | 72% | 84% |
| Person-days of accelerometer monitoring | 162 | 162 | 324 | 172 | 58 | 141 | 148 | 299 | 142 |
| Median % of Work Shift with accelerometer data | 100% | 100% | 100% | 100% | 100% | 100% | 100% | 100% | 100% |
| Person-days of HR monitoring | 162 | 162 | 324 | 172 | 58 | 141 | 148 | 299 | 142 |
| Median % of Work Shift with HR data | 100% | 100% | 100% | 97% | 99% | 100% | 100% | 93% | 94% |
| Person-days with self-reported hydration practices | 164 | 168 | 330 | 174 | 66 | 156 | 150 | 321 | 177 |

WBGT = wet bulb globe temperature; T_c_ = core body temperature; HR = heart rate

Table S2. Summary statistics for shift characteristics, work monitoring, and biomarker data, by job task within sugar industry

|  | El Salvador | | | | | | Nicaragua | |
| --- | --- | --- | --- | --- | --- | --- | --- | --- |
|  | Harvester | Sower | Driver/ Machine Operator | Supervis-or/ Irrigator | Agriche-mical Worker | Crop Maintenance | Harvester | Agriche-mical Worker |
| Number of person-days | 168 | 66 | 34 | 24 | 13 | 12 | 171 | 176 |
| Mean Work Shift Duration (SD) (hours) | 4.1  (2.6) | 4.1  (2.3) | 8.5  (1.3) | 8.2  (1.7) | 3.5  (0.4) | 2.7  (0.3) | 5.8  (1.2) | 3.0  (0.7) |
| Typical Shift Start Time (1^st^ quartile-3^rd^ quartile) | 6:20-  7:16 | 6:40-  7:00 | 6:41-  7:30 | 7:00-  7:35 | 6:49-  6:51 | 6:53-  7:08 | 6:51-  7:30 | 6:41-  7:42 |
| Typical Shift Stop Time (1^st^ quartile-3^rd^ quartile) | 8:00-13:52 | 9:56-10:29 | 15:48-16:00 | 16:00-16:05 | 10:00-10:30 | 9:35-10:00 | 11:45-14:15 | 9:11-10:45 |
| Median WBGT (MAD) (°C) | 26.9  (4.3) | 27.4  (1.5) | 27.8  (0.6) | 28.2  (0.8) | 25.5  (0.4) | 26.3  (1.0) | 27.1  (1.3) | 27.3  (1.3) |
| Median Heat Index (MAD) (°C) | 29.4  (6.0) | 31.5  (1.4) | 32.2  (1.0) | 32.4  (0.9) | 30.4  (0.4) | 29.1  (1.2) | 31.8  (1.5) | 31.0  (0.9) |
| Median T_c_ (MAD) (°C) | 37.8 (0.27) | 37.7 (0.23) | 37.5 (0.27) | 37.6 (0.27) | 37.5 (0.06) | 37.8 (0.21) | 37.9 (0.22) | 37.8 (0.37) |
| Maximum T_c_ (MAD) (°C) | 39.1 (0.27) | 38.8 (0.20) | 38.3 (0.32) | 38.2 (0.21) | 38.1 (0.14) | 38.2 (0.21) | 38.9 (0.19) | 39.8 (0.40) |
| Median Metabolic Rate (MAD) (kcal/hour) | 299.2 (77.7) | 131.3 (60.4) | 57.5 (31.4) | 57.9 (16.9) | 111.4 (77.2) | 159.2 (66.6) | 297.7 (52.1) | 317.5 (145.3) |
| Median % HR_max_ (MAD) | 60% (7%) | 50% (7%) | 46% (6%) | 45% (4%) | 48% (7%) | 51% (8%) | 62% (4%) | 62% (9%) |
| Median water consumption rate (MAD) (L/hour) | 1.2  (0.4) | 1.1  (0.6) | 0.5  (0.2) | 0.5  (0.2) | 1.2  (0.5) | 1.1  (0.3) | 1.3  (0.4) | 1.2  (0.4) |
| Median electrolyte consumption during shift (MAD) (L) | 0  (0) | 0  (0) | 0  (0) | 0  (0) | 0  (0) | 0  (0) | 1.6  (0.6) | 1.2  (0.3) |
| Median consumption of other liquids during shift (MAD) (L) | 0.3  (0.4) | 0.3  (0.4) | 0.3  (0.4) | 0.3  (0.4) | 0.3  (0) | 0.3  (0.2) | 1.3  (0.7) | 0.0  (0) |

MAD = Median Absolute Deviation

Table S3. Summary statistics for monitoring and biomarker data, by job task within non-agricultural industries.

|  | El Salvador | | | | Nicaragua | | | |
| --- | --- | --- | --- | --- | --- | --- | --- | --- |
|  | Construction | | | | Brick | | | |
|  | Driver/  Machine Operator | Machinery Assistant | Manual Assistant | Road Safety Worker | Carrier | Clay Worker | Clay Worker/  Carrier | Oven Worker |
| Number of person-days | 13 | 15 | 113 | 15 | 42 | 150 | 31 | 74 |
| Mean Work Shift Duration (SD) (hours) | 9.2  (0.7) | 9.9  (0.3) | 9.1  (1.2) | 9.4  (1.4) | 7.5  (4.2) | 6.2  (2.0) | 7.5  (2.7) | 10.4  (6.7) |
| Typical Shift Start Time (1^st^ quartile-3^rd^ quartile) | 7:00-  7:04 | 7:00-  7:05 | 7:00-  7:20 | 7:12-  7:30 | 3:33-  6:48 | 2:00-  4:00 | 2:03-  4:03 | 3:40-  9:40 |
| Typical Shift Stop Time (1^st^ quartile-3^rd^ quartile) | 16:00-16:53 | 16:45-  17:00 | 15:00-17:00 | 16:45-  17:38 | 10:40-16:39 | 8:35-10:40 | 9:50-11:07 | 7:25-  17:35 |
| Median WBGT (MAD) (°C) | 28.9  (0.7) | 28.9  (1.1) | 29.0  (0.7) | 29.0  (1.4) | 27.9  (1.0) | 24.9  (1.3) | 27.0  (1.9) | 28.1  (1.7) |
| Median Heat Index (MAD) (°C) | 32.8  (0.7) | 33.5  (2.2) | 33.2  (1.7) | 34.1  (2.0) | 32.5  (2.0) | 29.2  (1.7) | 29.9  (0.8) | 34.8  (2.7) |
| Median T_c_ (MAD) (°C) | 37.5 (0.09) | 37.5  (0.09) | 37.6 (0.17) | 37.4 (0.19) | 37.5 (0.27) | 37.6 (0.19) | 37.5 (0.21) | 37.3 (0.29) |
| Maximum T_c_ (MAD) (°C) | 38.2 (0.06) | 38.9  (0.16) | 40.2 (0.18) | 38.3 (0.23) | 39.1 (0.32) | 38.7 (0.23) | 38.4 (0.13) | 38.4 (0.26) |
| Median Metabolic Rate (MAD) (kcal/hour) | 58.9  (30.9) | 87.7  (75.8) | 97.4  (46.5) | 36.8  (18.0) | 157.9  (58.4) | 209.8  (61.9) | 209.9  (64.7) | 58.2  (76.9) |
| Median % HR_max_ (MAD) | 50%  (7%) | 51%  (2%) | 51%  (9%) | 48%  (4%) | 51%  (8%) | 53%  (8%) | 51%  (4%) | 49%  (6%) |
| Median Water consumption rate (MAD) (L/hour) | 0.4  (0.1) | 0.4  (0.0) | 0.4  (0.1) | 0.4  (0.1) | 0.5  (0.3) | 0.4  (0.2) | 0.3  (0.1) | 0.4  (0.3) |
| Median consumption of other liquids during shift (MAD) (L) | 0.3  (0) | 0.3  (0) | 0.3  (0) | 0.6  (0.4) | 0.8  (0.8) | 0.6  (0.6) | 0.9  (0.9) | 0.6  (0.4) |

MAD = Median Absolute Deviation

Table S4. Summary statistics for monitoring and biomarker data, by job task within non-sugar agricultural industries.

|  | El Salvador | | Nicaragua | | | |
| --- | --- | --- | --- | --- | --- | --- |
|  | Corn | | Plantain | | | |
|  | Agrichemical Worker | Harvester | Agrichemical Worker | Crop Maintenance | Harvester/  Crop Maintenance | Supervisor/  Irrigator |
| Number of person-days | 59 | 252 | 35 | 43 | 25 | 17 |
| Mean Work Shift Duration (SD) (hours) | 1.6  (0.7) | 3.5  (1.3) | 9.6  (2.6) | 6.2  (2.5) | 8.9  (2.5) | 10.5  (7.1) |
| Typical Shift Start Time (1^st^ quartile-3^rd^ quartile) | 6:56-  7:55 | 5:50-  6:10 | 6:10-  6:25 | 6:25-  6:38 | 6:24-  6:45 | 6:30-  6:44 |
| Typical Shift Stop Time (1^st^ quartile-3^rd^ quartile) | 8:41-  9:20 | 8:30-  10:00 | 17:00-  17:00 | 11:07-  12:00 | 13:00-  17:00 | 10:41-  16:50 |
| Median WBGT (MAD) (°C) | 26.0  (2.5) | 27.1  (2.2) | 29.3  (1.8) | 29.0  (1.8) | 29.2  (1.3) | 29.9  (0.9) |
| Median Heat Index (MAD) (°C) | 27.1  (3.9) | 30.3  (3.6) | 35.0  (0.2) | 34.8  (2.9) | 35.4  (1.0) | 36.3  (1.2) |
| Median T_c_ (MAD) (°C) | 37.4  (0.25) | 37.4 (0.28) | 37.4  (0.32) | 37.6  (0.30) | 37.7  (0.10) | 37.4  (0.24) |
| Maximum T_c_ (MAD) (°C) | 38.4  (0.23) | 38.9 (0.40) | 37.9  (0.20) | 39.2  (0.43) | 38.2  (0.15) | 38.4  (0.26) |
| Median Metabolic Rate (MAD) (kcal/hour) | 179.2  (77.8) | 88.2  (41.4) | 47.6  (47.2) | 173.1  (97.8) | 124.8  (80.6) | 39.8  (38.0) |
| Median % HR_max_ (MAD) | 55%  (6%) | 54%  (6%) | 47%  (9%) | 50%  (10%) | 45%  (5%) | 42%  (4%) |
| Median water consumption rate (MAD) (L/hour) | 1.9  (1.2) | 0.9  (0.4) | 0.4  (0.1) | 0.6  (0.2) | 0.6  (0.2) | 0.4  (0.1) |
| Median consumption of other liquids during shift (MAD) (L) | 0.3  (0) | 0.2  (0.2) | 0.6  (0.4) | 0.3  (0.4) | 0.5  (0.7) | 0.5  (0.3) |

MAD = Median Absolute Deviation
